# Mesenchymal stromal cell-derived exosomes improve pulmonary hypertension through inhibition of pulmonary vascular remodeling

**DOI:** 10.1186/s12931-020-1331-4

**Published:** 2020-03-20

**Authors:** Shanshan Zhang, Xiaoli Liu, Li Li Ge, Kailin Li, Yongchao Sun, Fang Wang, Ying Han, Chao Sun, Jue Wang, Wen Jiang, Qian Xin, Chaoyue Xu, Yuan Chen, Ou chen, Zhaohua Zhang, Yun Luan

**Affiliations:** 1 The Second Hospital of Shandong University, No. 247, Beiyuan Dajie, Jinan, 250033 People’s Republic of China; 2 Department of Hematology, The Second Hospital of Shandong University, Jinan, People’s Republic of China; 3 Institute of Biotherapy for Hematological Malignancies, The Second Hospital of Shandong University, Jinan, People’s Republic of China; 4 Department of Special Inspection, The Second Hospital of Shandong University, Jinan, People’s Republic of China; 5 Institute of Medical Science, Central Research Laboratory, The Second Hospital of Shandong University, No. 247, Beiyuan Dajie, Jinan, 250033 People’s Republic of China; 6 Department of Medicine, Jinan Vocational College of Nursing, Jinan, People’s Republic of China; 7 Institute of Medical Science, Animal center, The Second Hospital of Shandong University, Jinan, People’s Republic of China; 8 Department of Pediatrics, The Second Hospital of Shandong University, Jinan, People’s Republic of China; 9 School of nursing, Shandong University, Jinan, People’s Republic of China

**Keywords:** Pulmonary hypertension, Exosomes, Pulmonary vascular remodeling, Wnt5a

## Abstract

**Background:**

Pulmonary hypertension (PH) is a life-threatening disease characterized by pulmonary vascular remodeling, right ventricular hypertrophy and failure. So far no effective treatment exists for this disease; hence, novel approaches are urgently needed. The aim of the present research was to observe the treatment effect of mesenchymal stromal cell derived exosomes and reveal the mechanism.

**Methods:**

Monocrotaline (MCT)-induced PH in rats and hypoxia-induced cell damage model were established, respectively. Exosomes derived from the supernatant of human umbilical cord mesenchymal stem cells (MSC-exo) were injected into MCT-PH model rat or added into the cells cultured medium. Immunohistochemistry, quantitative real-time polymerase chain reaction (qRT-PCR) and western blot methods were used in vivo and *vitro*.

**Results:**

The results showed that MSC-exo could significantly attenuate right ventricular (RV) hypertrophy and pulmonary vascular remodelling in MCT-PH rats. In the cell culture experiments, we found that MSC-exo could significantly inhibit hypoxia-induced pulmonary arterial endothelial cell (PAEC) apoptosis and pulmonary arterial smooth muscle cells (PASMC) proliferation. Furthermore, the pulmonary arterioles endothelial-to-mesenchymal transition (EndMT) was obviously suppressed. Moreover, the present study suggest that MSC-exo can significantly upregulate the expression of Wnt5a in MCT-PH rats and hypoxic pulmonary vascular cells. Furthermore, with Wnt5a gene silencing, the therapeutic effect of MSC-exo against hypoxia injury was restrained.

**Conclusions:**

Synthetically, our data provide a strong evidence for the therapeutic of MSC-exo on PH, more importantly, we confirmed that the mechanism was associated with up-regulation of the expression of Wnt5a. These results offer a theoretical basis for clinical prevention and treatment of PH.

## Background

Pulmonary hypertension (PH), a pathological complex circulation disease, is characterized by pulmonary vascular remodeling, right ventricular hypertrophy and failure [1, 2]. Gradually increased pulmonary vascular resistance eventually lead to the right ventricle (RV) hypertrophy and failure [3, 4]. The vascular remodeling including dysfunction of pulmonary arterial endothelial cell (PAEC), excessive proliferation of pulmonary arterial smooth muscle cells (PASMC), distal pulmonary arterioles muscularization, extracellular matrix protein deposition, and inflammation [5, 6]. At present, no effective method to treat PH vascular remodeling, new treatments are urgently needed.

Work by our group and others have showed that intravenous delivery of mesenchymal stem/stromal cell (MSC) could improve experimental PH vascular remodeling and right ventricular impairments [7, 8], however, the mechanism is not very clear. MSC-derived exosomes (MSC-exo) is one of the main therapeutic vectors in MSCs, which act as vehicles transmission of protein species in the cells directly affect the receptor cells gene expression and cell phenotype. Several studies have shown that that intravenous delivery of MSC-exo can inhibit PH vascular remodeling [9–11], however, the molecular mechanism is still not clear.

Wnt5a is one of a member of the Wingless (Wnt), activates non-canonical or canonical Wnt signaling pathways through specific coupling of different receptor. Reports showed that down-regulation of Wnt5a expression could markedly increase hypoxia-induced PASMC proliferation [12, 13], but loss of it could reduce the formation of new vessels resulting in PH [14]. Thus, the production of Wnt5a is likely to become a new way to prevent the small vessel loss in PH.

Here, our results demonstrated that MSC-exo could up-regulation the expression of Wnt5a in MCT-PH rats lung tissue and hypoxia-induced cells, therefore, we hypothesized that MSC-exo could improve PH injury through regulating Wnt5a signaling pathway.

## Materials and methods

### Animal model and hypoxic exposure

All animals received humane care in compliance with the *Guide for* the *Care and Use of Laboratory Animals* published by the US National Institute of Health. Also, all experiments were approved by the Institutional Animal Care and Use Committee of Shandong University. The monocrotaline-induced PH (MCT-PH) rat model has been well-established extensively in our previous studies [15, 16]. After study, all animals were anaesthetized by isoflurane inhalation (1.5–2%) and then euthanized by cervical dislocation. Rat PAMSC and PAEC hypoxic damage model was established under hypoxia (3%) in the presence or absence of exosomal fraction (100 μg/ml).

### Isolation and culture of hUCMSC

We collected an umbilical cords sample from disconnected umbilical cords following full-term normal delivery, there was no history of maternal chorioamnionitis and /or other active infectious processes. The experiment was carried out with the written informed consent from the recruited adult and the newborns’ parents. MSC from umbilical cord Wharton’s Jelly was isolated as previously described with some modification [10]. The umbilical cord was rinsed with phosphate-buffered saline (PBS), removed the vessels, and then cut into 1–2 mm^3^ pieces and digested for 60 min anenzyme (Sigma, St. Louis, MO, USA) at 37 °C in a shaking incubator. The immunotyping characterization of hUCMSCs occurred at passage 3–4 using human specific antibodies CD34, CD45, CD73, CD90, CD105, HLA-DR (BD Biosciences Pharmingen, San Diego, CA) by a fluorescence-activated cell sorter (FACS, BD FACSAria II). The capacity of osteogenesis and adipogenic differentiation of MSC were performed by special medium. The plates were seen using an inverted microscope, and images were taken from them.

### Preparation of exosomes

When reached 90% confluences, the adherent cells were incubated in culture medium with 5% exosome-depleted fetal bovine serum (FBS) for 24 h, and 5–8 passages human umbilical cord MSC (hUCMSCs) were used for experiments. The conditioned medium was centrifuged at 4 °C at 300 g for 10 min at 2000 g for 10 min and finally at 10000 g for 30 min to remove the cells and debris, followed by centrifugation of the supernatant at 100000 g at 4 °C for 1 h, The hUCMSC derived exosomes (MSC-exo) were resuspended in PBS and filtered with a 0.22 μm microfiltration membrane, centrifuged again in PBS at 100,000 g for 1 h to collect the exosomes, which were re-suspended in PBS stored at − 80 °C. The protein concentration was determined using a bicinchoninic acid (BCA) assay kit, and labeled with PKH26 Red Fluorescent Cell Linker Mini Kit (Sigma) according to the manufacturer’s protocol. MSC-exo The morphology of MSC-exo was examined using a transmission electron microscope (TEM) according to the manufacturer’s instructions. Briefly, the prepared exosomes were stained with phosphotungstic acid solution and then performed under a Hitachi-9000 TEM system. The expression of CD63, CD81, TS101 and Alix was confirmed by Western blot.

### Monocrotaline (MCT)-induced PH models

PH model was established by a single intraperitoneal injection of MCT(60 mg/kg; Sigma, St. Louis, MO, USA) in adult male Sprague-Dawley rats as described previously [17, 18]. Three weeks after the MCT injections, the animals received were given at a dose of 25 μg/day or an equal volume of MSC-culture medium (MSC-CM) via tail vein injection once daily for 3 days as previously study [9], the protein concentration of MSC-exo was determined by a bicinchoninic acid (BCA) assay kit, and 25 μg protein in 100 μl PBS was used.

Animals were anesthetized with pentobarbital (50 mg/kg) and injected through the left jugular vein with concentrated conditioned media or exosome preparations. The animals were evaluated at 4 weeks after MCT administration, rats (*n* = 20) were randomly assigned to 4 groups (*n* = 5 in each group). Hemodynamic data were recorded post-operation as previously described with some modifications. Briefly, after reanesthetization, rats were ins inserted with a 3-Fr Miller catheter via the right jugular vein into the right ventricle to obtain measurements of right ventricular systolic pressure (RVSP). The rats were then euthanized and the hearts were harvested, the right ventricle (RV) was separated from the left ventricle (LV) plus septal wall (S), and both parts were weighed to assess hypertrophy (RVH). The ratio of right ventricular free wall to left ventricular plus septal weight (RV/LV + S) was determined to measure the right ventricular hypertrophy.

### Immunological and immunoistochemical analyses of pulmonary vessels and cardiomyocytes

After hemodynamic data were recorded, the hearts and lungs were quickly harvested, fixed in 4% paraformaldehyde and embedded in paraffin, the serially sectioned at a thickness of 4–5 μm were stained with hematoxylin and eosin (H&E) and Masson’s trichrome, respectively. Muscularization of pulmonary vessels was counted as previously study [19]. Images of RV cardiomyocytes were captured and analyzed using the Olympus automatic analysis system (Olympus Corporation, Japan). The average of the 10 high-power fields (hpf) was randomly selected, and positively stained areas were padded with a single color and converted into pixels through optical density (OD) calibration. The percent of muscular artery (MT%) with an external diameter of 15 to 50 μm was used to evaluate medial thickening of pulmonary arterioles.

To investigate the effects of MSC-exo on pulmonary arterioles, the expression of smooth muscle cell marker a-SMA was measured by immunofluorescence staining to quantify the muscularization of vessels. Briefly, 4-5 μm-thick cryosections were first blocked with 5% goat serum for 30 min. The sections were then incubated with a-SMA (ab21027) or a nonspecific IgG antibody for 1 h at room temperature, which was followed by 1-h incubation in the dark with secondary antibody. Nuclei were stained with 4,6-diamidino-2-phenylindole (DAPI, Sigma-Aldrich). Fluorescent images were taken with a Nikon Eclipse 90i microscope.

### Cell culture and hypoxia-induced preparation

Rats PAEC and PAMSC were purchased from Procell Life Science&Technology Co.,Ltd. Wuhan, China. PAECs were cultured in M200 complete medium supplemented with low serum growth supplement (LSGS, Invitrogen), or smooth muscle growth medium supplemented with smooth muscle growth supplement (SMGS; Cascade Biologics) with 100 Ug/ml of penicillin, 100 IU/ml streptomycin. Medium was changed every 2 to 3 days, confluence cells (> 80%) were digested with 0.05% trypsin including 0.04% ethylenediamine-tetraacetic acid (Sigma-Aldrich, St. Louis, MO) in PBS. Endothelial cells marker CD31 (AF3628) and smooth muscle cells markers α-SMA (ab21027) were detected to identify the cells Passage 3–10 cells were used in all experiments. Hypoxic cell damage model was established as the previous report [20], briefly, before exposure to hypoxia, cells were incubated in serum-free medium for 24 h and then cells were cultured in a hypoxia incubator (Thermo Electron, Forma, MA) under hypoxia (3% O_2_, 5% CO_2_, 92% N_2_) or normoxia condition (21% O_2_, 5% CO_2_,74% N_2_) that was maintained at 37 °C in the presence or absence of MSC-exo fraction (100 μg/ml), or the exosome-depleted fraction of culture medium (MSC-CM), respectively. Cell growth was arrested under normoxia or hypoxia for 24 h, 48 h and 72 h.

### siRNA targeting Wnt5a

To investigate the role of wnt5a in MSC-exo to hypoxia-induced lung cells injury protection role, wnt5a was knocked down in PASMC and PAEC by transfecting siRNA targeting wnt5a. siRNA oligonucleotides with two thymidine residues (dTdT) at the 3′ end of the sequence were purchased from GenePharma (GenePharma, Shanghai, China). The siRNA oligonucleotides were selected to correspond to thenucleotide sequence of si-r-Wnt5a: 5′-GGACAACACTTCTGTCTTT-3′.Total RNA was extracted from cells using a Qiagen RNeasy kit (Qiagen, Basel, Switzerland). Complementary DNA (cDNA) first strand was produced using a Superscript first-strand synthesis system using oligo (dt) antisense primers (Invitrogen, Lucerne, Switzerland). Amplified fragments were analyzed in 1.5% agarose gel electrophoresis in the presence of ethidium bromide (Sigma-Aldrich). GAPDH was used as an internal control for the amount of RNA input.

### Cell’s immunofluorescence to analyze Wnt5a levels

We used cell’s immunofluorescence to analysis the Wnt5a levels in PAEC and PAMSC, briefly, cells were incubated with anti-Wnt5a antibody (ab238422) or stained with anti-CD31 (AF3628) and anti-α-SMA (ab21027) antibodies at 37 °C for 1 h, then secondary antibody for 2 h. Nuclei were stained with 4, 6-diamidino-2-phenylindole (DAPI, Sigma-Aldrich). Fluorescence images were captured by use of the ZEISS 800 confocal system. All sections were examined and at least three to five images from each section were acquired.

### Cells proliferation and apoptosis assay in vitro

PASMC proliferation was measured using the BrdU Cell Proliferation Assay Kit (Sigma-Aldrich). For BrdU incorporation assays, cells were plated in 96-well plates at a density of 5 × 10^3^ cells/well in culture medium with 10% FBS under standard culture conditions. After confluence > 80%, they were incubated in normoxia or 3% oxygen for 0 h, 24 h,48 h and 72 h treatment with MSC-CM or MSC-exo, and then they were incubated with 5-BrdU labeling solution for another 2 h. Detection antibody and HRP-conjugated secondary antibody were added, absorbance of BrdU plates was measured at 450 nm. PAEC apoptosis were detected using Annexin V-FITC/PI apoptosis detection kit (Roche Diagnostics, Indianapolis, IN, USA) according to the manufacturer’s instructions as our previously reported [21]. Briefly, 1 × 10^6^ cells treatment with MSC-CM or MSC-exo and then incubated in normoxia or 3% oxygen for 48 and 72 h, they were collected and suspended in 500 μl binding buffer, 5 μl Annexin V-FITC and 5 μl PI were added to each sample and incubated for 15 min in the dark. The percent of apoptosis were analysis by FACScan flow cytometry (FACS LSRFortessa; BD Biosciences, Franklin Lakes, NJ, USA). Triplicate experiment with triplicate samples were performed.

### In vitro tube formation on Matrigel-coated plates

We seeded PAECs on Matrigel formed capillary-like cellular network, cultured the cells in a normoxia or 3% oxygen condition in the presence or absence of MSC-exo for 72 h, and then examined the effects of MSC-exo on tube formation ability with an in Matrigel (Corning Inc., Tewksbury, MA, USA) model. Hence, the cells aggregated to form cell cords that jointed each other and/or branched into a network. Briefly, The solution was added into the wells and incubated at 37 °C for 1 h to allow matrix gel solidification. The PAEC in each group were re-suspended in EBM-2 basic medium at 10^5^ per ml. Continue to cultivate cells for 4 h, capillary-like tubular structures were photographed under a Nikon Eclipse 90i microscope (Nikon, Tokyo, Japan) and five representative fields from each well were photographed. The branch points of capillary structure were counted.

### Quantitative real-time polymerase chain reaction to analyze apoptosis genes

Total RNA was extracted with Trizol reagents from lung tissue and cells using a Qiagen RNeasy kit (Qiagen, Basel, Switzerland). Complementary DNA (cDNA) first strand was produced using a Superscript III cDNA synthesis kit (Bio-Rad, Hercules, CA) using oligo (dt) antisense primers (Invitrogen, Lucerne, Switzerland). Quantitative real-time polymerase chain reaction (qRT-PCR) analysis was performed using a M × 3000P System.1.5% agarose gel electrophoresis in the presence of ethidium bromide (Sigma-Aldrich) was used to amplification fragments, and β-actin as and internal control. Specific primers used for sequence: Bcl-2 (Forward 5’AGAGGGGCTACGAGTGGGAT3’, Reverse 5’CTCAGT CATCCACAGGGCGA-3′), Bax (Forward 5’GGTTTCATCCAGGATCGAGACG3’, Reverse 5′ ACAAAGATGGTCAGGGCTTGCC3’), Caspase-3 (Forward5’CGTGCAGTC AACTGCCGCAAGA3’, Reverse 5’CCGGGTCACAGGCCAGGTATG3’), β-5’CTCTTTG ATGTCACGCACGATTTC3’, Reverse 5’GTGGGCCGCTCTAGGCACCAA3’).

### Western blot to analyze protein expression

The tissues and cells protein concentration was detected using a BCA assay kit, lysates were separated by polyacrylamide gel electrophoresis (PAGE) and electro-transferred onto a polyvinylidene fluoride (PVDF), The embranes were blocked in 5% skimmed milk-Tris-buffered saline plus Tween-20 solution and incubated with primary antibodies of CD63(Invitrogen,10628D), CD81(MA5–32333), TSG101(MA5–32463), ALIX(ab186429), CD31(AF3628), α-SMA (ab21027), Wnt5a (ab174963), β-catenin (ab32572), Cyclin D1(MA5–15512), PCNA(ab92552), GSK3β (ab93926) and RhoA (ab219371), respectively, overnight at 4 °C. The primary antibody-labeled membranes were then treated with the horseradish peroxidase (HRP)-conjugated goat anti-rabbit secondary antibody to IgG (ab205718) at room temperature for 1.5 h. GAPDH or β-actin expression was used as an internal control.

### Statistical analysis

Data of continuous variables are presented as mean ± standard deviation (SD); while data not conforming to homogeneity of variance or normal distribution were expressed as interquartile range. Comparisons of mean values between two groups were analyzed using a non-paired t-test. Comparisons among multiple groups were analyzed by one-way analysis of variance (ANOVA), followed by Scheffe post hoc test. Statistical analysis was carried out by using the SPSS 1.9 software (IBM, Armonk, NY, USA). *P* < 0.05 was regarded as significant statistical difference.

## Results

### Characterization and differentiation potential of hUCMSCs

**Fig. 1:**
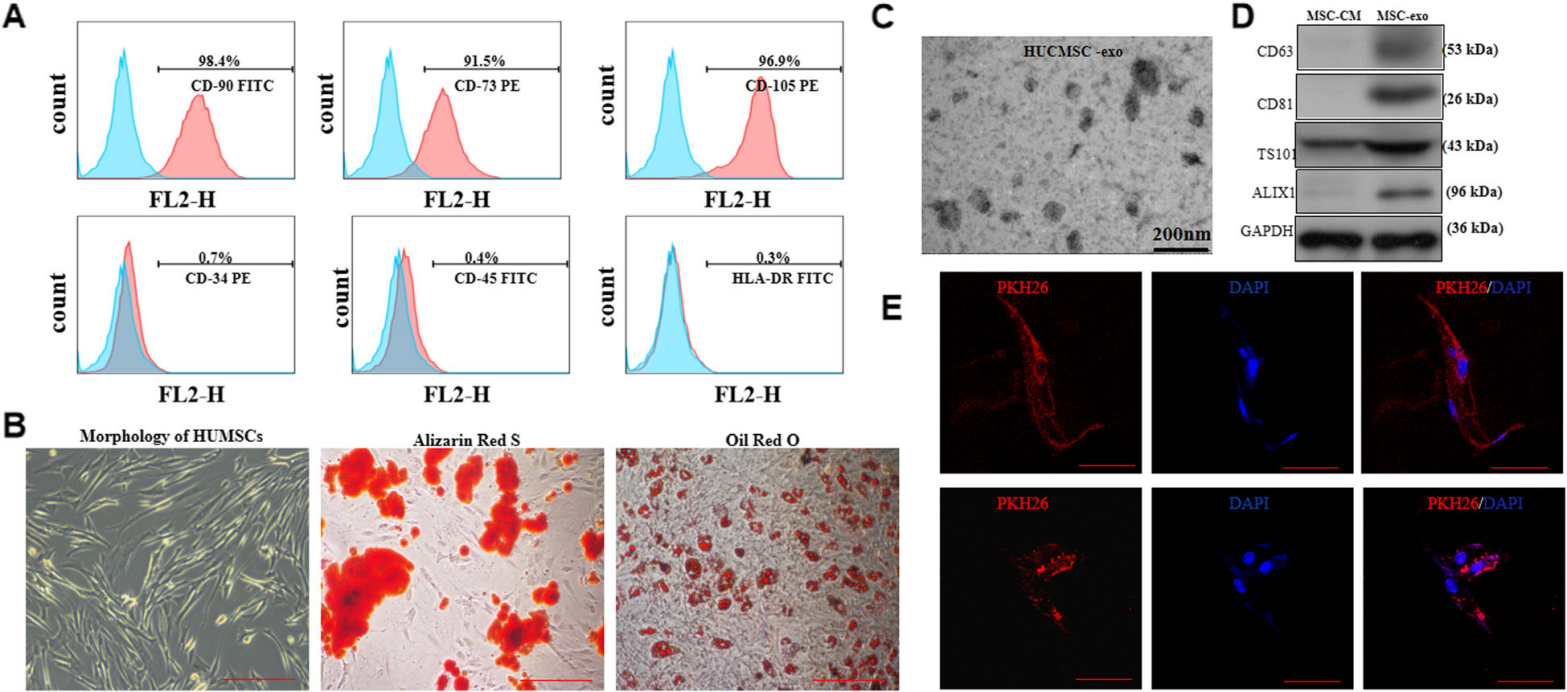
Characterization and differentiation of human umbilical cord MSCs and MSC-exo. **a** MSC surface marker expression analysis by flow cytometry. **b** Morphology and differentiated of MSCs into adipocytes and osteocytes. **c** Transmission-electron microscopy study of the shape of MSC-exo. **d** The protein content of MSC-exo analyzed by western blot. **e** PKH-26 labeling of MSC-exo up-taken by pulmonary arterial endothelial cells. Scale bar = 100 μm

As showed in Fig. 1a, flow cytometric analysis demonstrated that hUCMSCs demonstrated that the majority of MSCs expressed high levels of the CD73, CD90, and CD105 markers, whereas CD34, CD45 and HLA-DR markers were relatively absent. The Alizarin red and oil red O staining results showed that the hUMSCs had the ability to differentiate into osteocytes and adipocytes (Fig. 1b).

### MSC-exo characterization

The size and metabolic protein of MSC-exo derived from hUCMSC were evaluated by TEM imaging and western blot, as shown in Fig. 1c and d, MSC-exo range between 50 and 150 nm in size, and an enrichment of CD63, CD81, TSG101 and ALIX levels. The uptake of MSC-exo by the PAEC was observed under a confocal fluorescence microscopy at 24 h and 48 h after co-culture as shown in Fig. 1e, indicated that the intensity of cells fluorescent shows the rate of MSC-exo uptake by cells.

### MSC-exo attenuates monocrotaline-induced pulmonary vascular remodeling and right ventricle damage

**Fig. 2:**
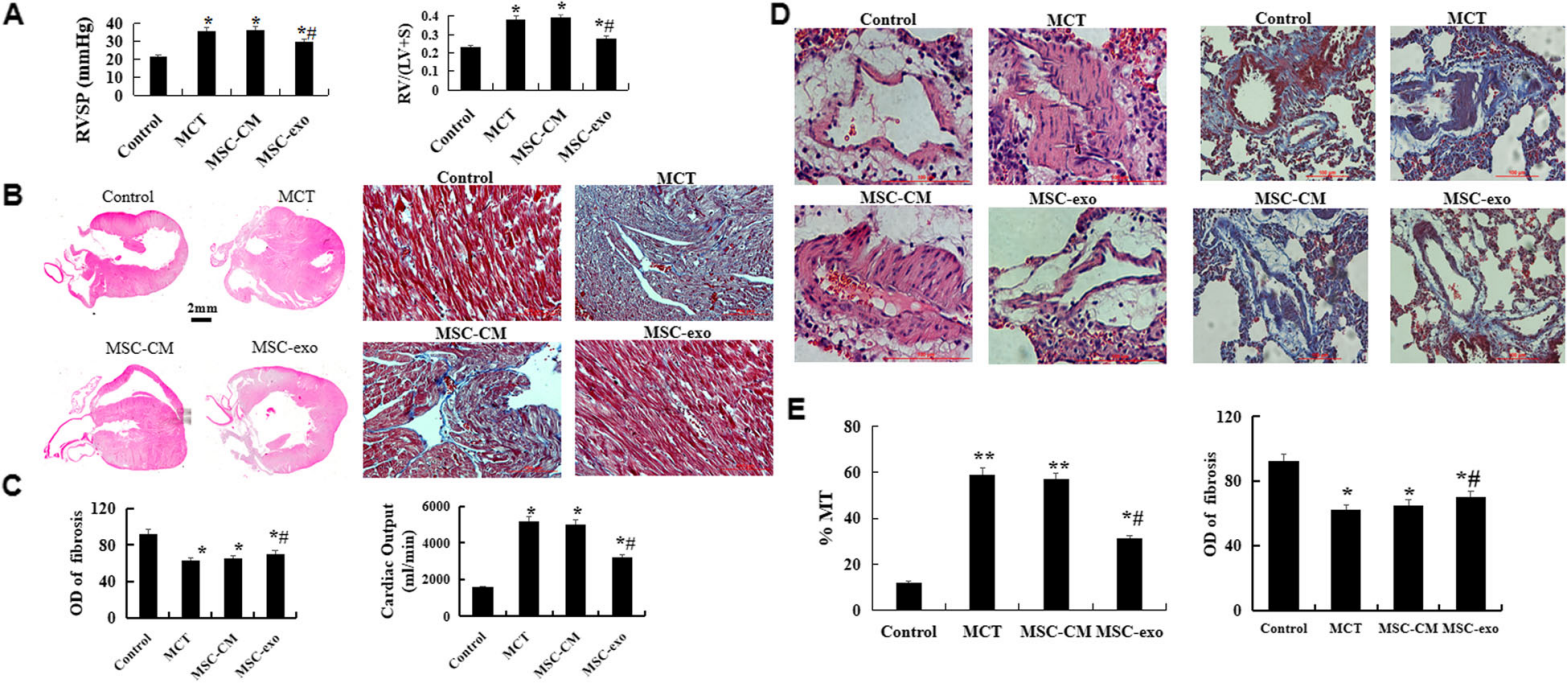
Effect of MSC-exo MCT-induced pulmonary artery pressure, vascular remodeling and right ventricle hypertrophy. **a** Comparative analysis of RVSP and RV/(LV + S) ratios (**b**) Morphology and histology of the heart were observed by hematoxylin and eosin (HE) and Masson’s staining. **c** Comparative analysis of fibrosis and cardiac output. **d** Pulmonary arterioles wall thickness and fibrosis were observed by HE and Masson’s staining. **e** Comparative analysis of the percent of muscular artery (MT%) and optical density (OD). *n* = 5 rats per group; *P* < 0.05, t-test; the data are present as mean ± SD; ^*^MCT or ^*^MSC-CM vs. control; ^#^MSC-exo vs. MCT group; scale bar = 100 μm

The results showed that the ventricular systolic pressure (RVSP) and right ventricle/left ventricle plus septum (RV/LV + S) ratio were significant decreased in MSC-exo group than that in MCT and MSC-CM groups (Fig. 2a, *P* < 0.05). H&E and Masson’s trichrome staining results showed that MSC-exo administration could significantly attenuate the the vessel wall thickness, right ventricular hypertrophy, the percent of muscular artery (MT%) with an external diameter of 15 to 50 μm, and the degree of fibrosis as compared with MCT and MSC-CM rats (*P* < 0.05, Fig. 2b-e).

### Effects of MSC-exo on MCT-induced EndMT andWnt5a signaling in rats

**Fig. 3:**
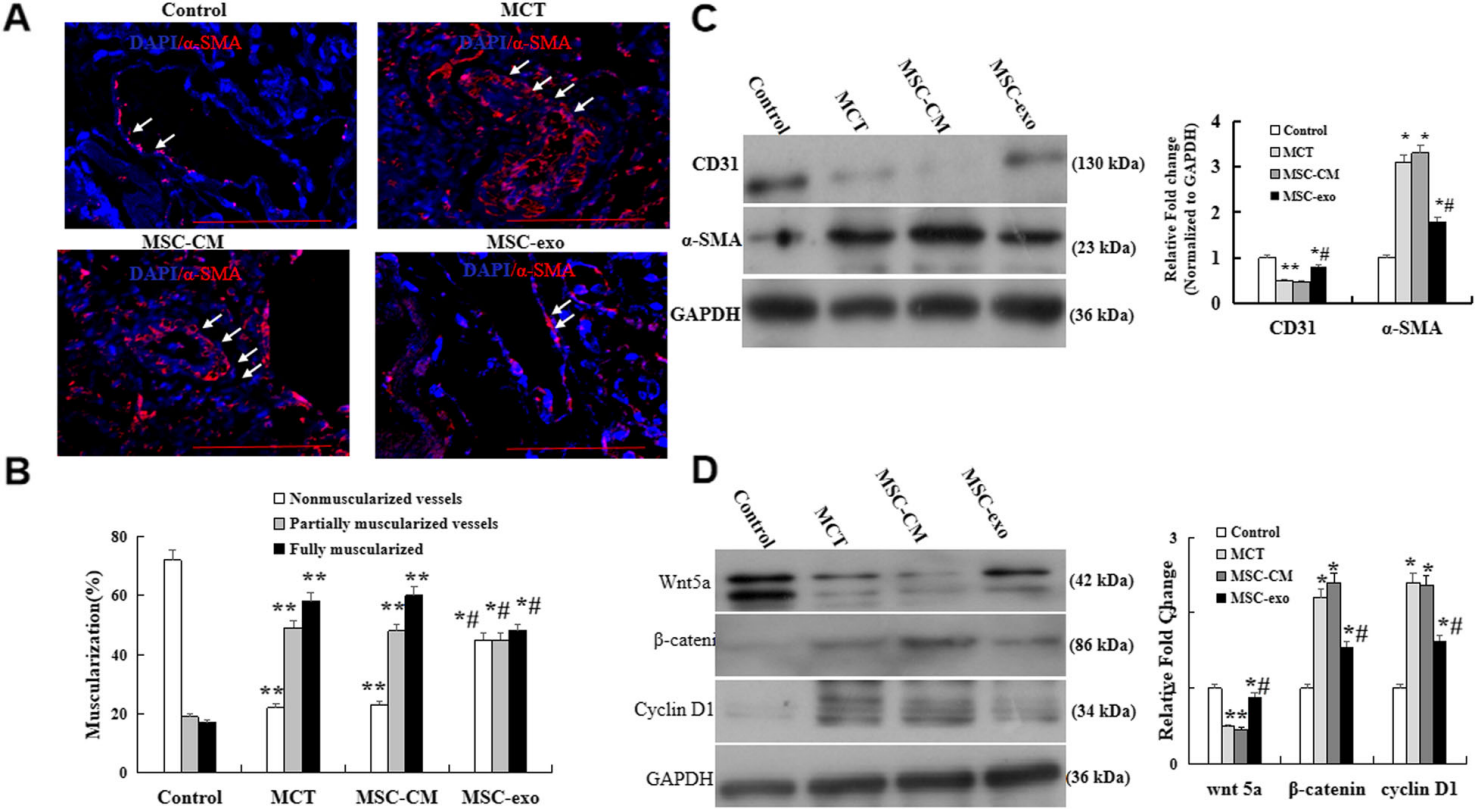
Effects of MSC-exo on MCT-induced endothelial-to-mesenchymal transition. **a** Representative immunofluorescence staining with a-SMA (Red) and the cell nuclei were labeled with DAPI (blue) in the pulmonary arterioles using a light microscope at a × 400 magnification. **b** Comparative analysis of non-muscularized vessels, partially muscularized vessels, and fully muscularized vessels. **c** Protein expression of CD31 and a-SMA analysis by western blot in lungs. **d** Detection and comparative analysis of the protein expression of Wnt5a, β-catenin and cyclin D1. *n* = 5 rats per group; *P* < 0.05, t-test; the data are present as mean ± SD; ^*^MCT or ^*^MSC-CM vs. control; ^#^MSC-exo vs. MCT group; scale bar = 100 μm

To investigate the effects of exosomes on MCT-induced pulmonary arterioles endothelial-to-mesenchymal transition (EndMT), the expression of ECs markers CD31 and smooth muscle cells markers α-SMA were detected in lung tissue by immunofluorescence and western blot. The results showed that the expression of α-SMA were evidently increased in MCT and MSC-CM groups, but a significant decreased in MSC-exo group (Fig. 3a). Quantitative analysis of the representative muscularization of peripheral pulmonary vessels demonstrated that the percentage of fully muscularized vessels was obviously reduced, but the proportion of non-muscularization was significantly increased in MSC-exo group than that in MCT and MSC-CM groups (*P* < 0.05, Fig. 3b). The western blot indicated that the expression of CD31 was significantly higher in MSC-exo group as compared with MCT or MSC-CM group (*P* < 0.05, Fig. 3c).

To determine the effect of MSC-exo on Wnt5a pathway molecules, the protein expression levels of wnt5a, β-catenin and cyclin D1 were detected by western blot. We found that the protein level of Wnt5a was significantly decreased, but β-catenin and cyclin D1 levels were increased in MCT and MSC-CM groups than that in control group, however, these results were reversed, shown as wnt5a expression higher, β-catenin and cyclin D1 expression lower as compared with MCT and MSC-CM groups (*P* < 0.05, Fig. 3d).

### Effect of MSC-exo on hypoxia-induced Wnt5a expression changes in vitro

**Fig. 4:**
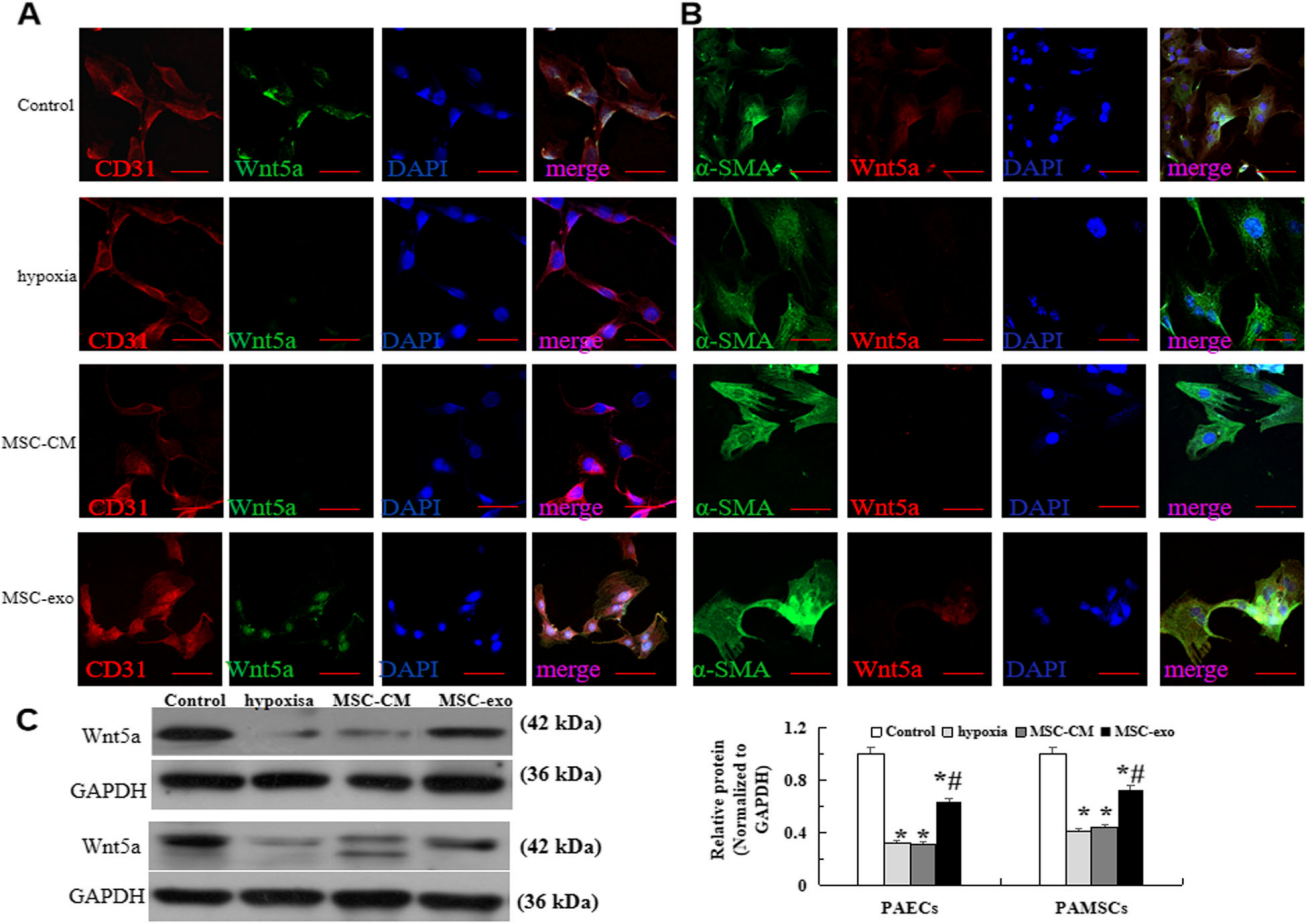
Wnt5a levels detecting in hypoxia induced PAECs and PAMSCs. **a** Immunofluorescence staining with CD31 (red), Wnt5a (green) and the cell nuclei were labelled with DAPI (blue) in the PAECs at a × 400 magnification. **b** Immunofluorescence staining with a-SMA (green), Wnt5a (red) and DAPI (blue) in PAMSCs at a × 400 magnification. **c** Protein expression and comparative analysis of of CD31 and a-SMA by Western blot. *n* = 3 times repeated; *P* < 0.05, one-way ANOVA followed by post hoc test; the data are present as mean ± SD; ^*^hypoxia or ^*^MSC-CM vs. control; ^#^MSC-exo vs. hypoxia group. Scale bar = 100 μm

In the present study, we used immunofluorescence and western blot to analysis the effect of MSC-exo on the expression of Wnt5a in high oxygen damage model, the results showed that when the cells were hypoxia exposure for 72 h, the expression of Wnt5a were significantly decreased in hypoxia-induced PAEC and PAMSC, this reduction was restored in MSC-exo group (*P* < 0.05, Fig. 4a-c).

**Fig. 5:**
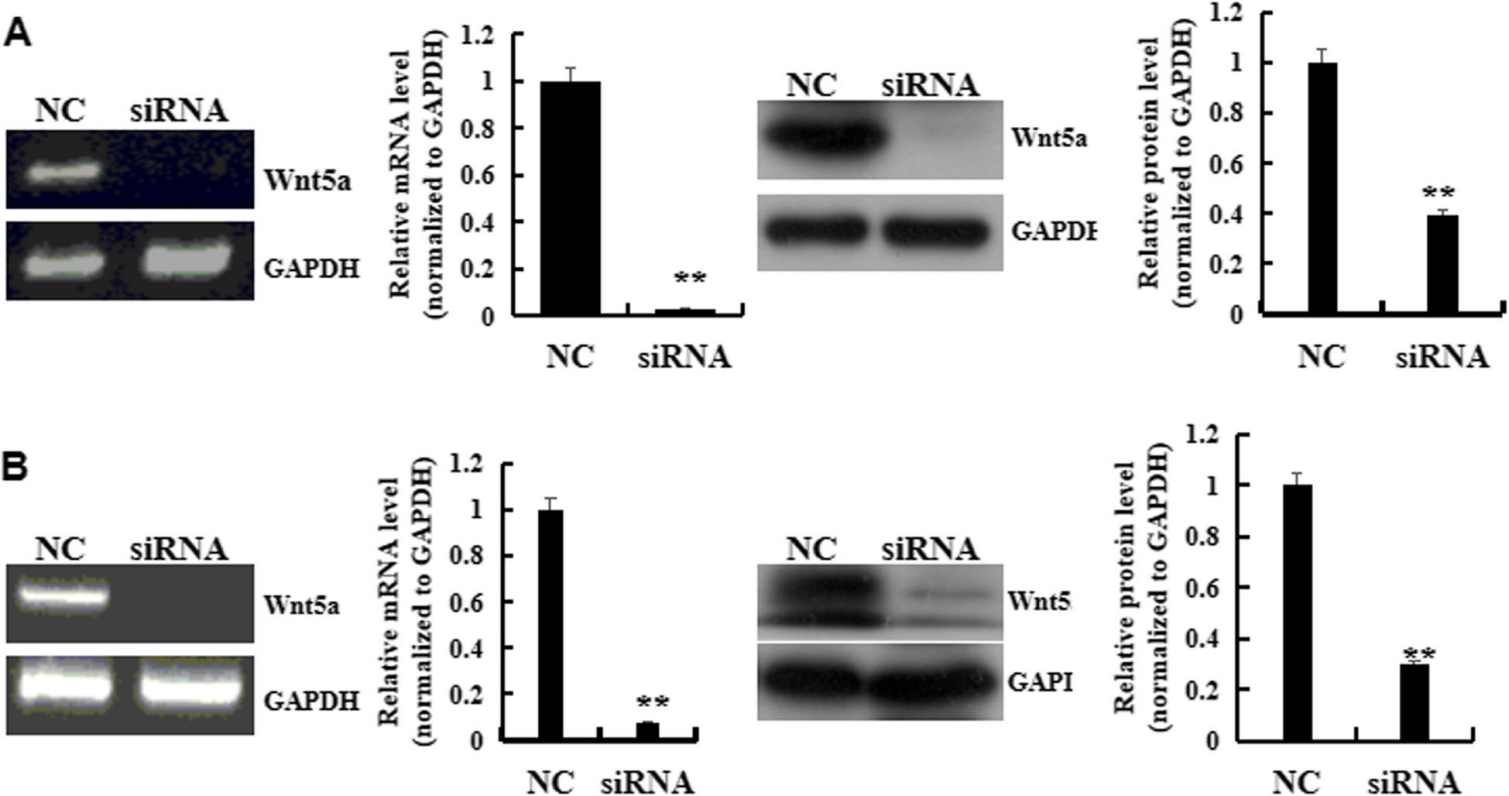
siRNA Wnt5a targeting transfecting in PAECs and PAMSCs. **a** PAECs. **b** PAMSCs. *n* = 3 times repeated; *P* < 0.01, t-test; the data are present as mean ± SD; ^**^siRNA vs. Normal

To investigate the role of Wnt5a pathway in MSC-exo against hypoxia-induced cell damage, wnt5a was knocked down by transfecting siRNA targeting Wnt5a. The results showed that transfection of wnt5a siRNA caused a reduction in Wnt5a expression and the inhibition efficacy was about 97.4 and 92.8% at mRNA level at mRNA level, 50.8 and 70.4% at protein level in PAEC and PAMSC, respectively (Fig. 5).

### Effect of MSC-exo on hypoxia-induced PAECs apoptosis in vitro

**Fig. 6:**
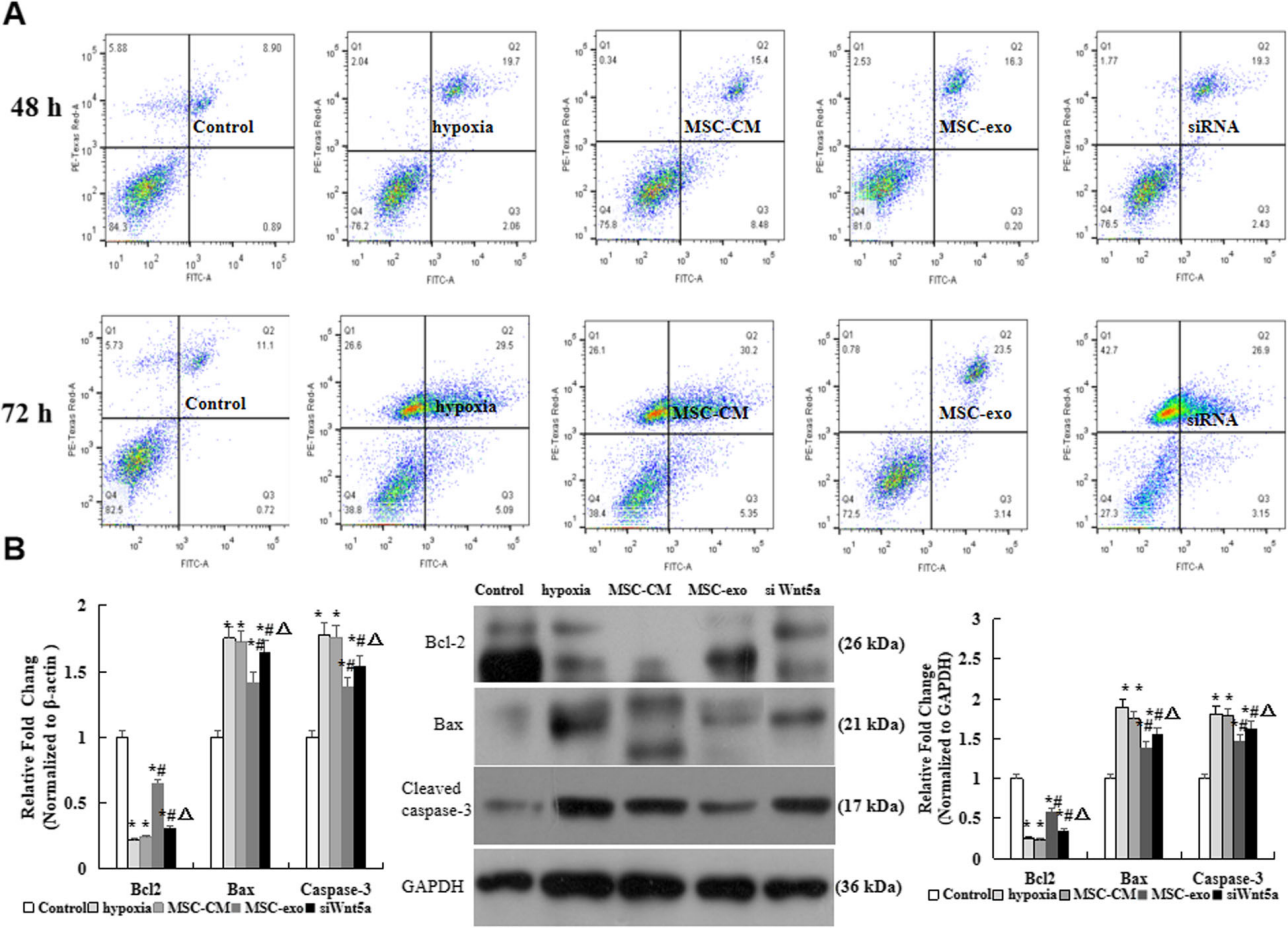
Effect of MSC-exo on hypoxia-induced PAECs apoptosis in vitro. **a** PAECs were stained with Annexin V/FITC and analyzed by flow cytometry when hypoxic exposure for 48 h and 72 h. **b** mRNA and protein expression levels of Bcl2, Bax and caspase-3 by RT-PCR abd Western blot in lungs. *n* = 3 times repeated; *P* < 0.05, one-way ANOVA followed by post hoc test; the data are present as mean ± SD; ^*^hypoxia or ^*^MSC-CM vs. control; ^#^MSC-exo vs. hypoxia group; ^*^siWnt5a vs. MSC-exo group

We detected cells apoptosis to observe the protection of MSC-exo on hypoxia induced PAEC damage. Briefly, after hypoxic exposure for 48 h and 72 h, as shown in Fig. 6a, the percentage of apoptotic cells was gradually increased in hypoxia group when compared with control group, however, the percentage was decreased in MSC-exo than hypoxia and MSC-CM groups (*P* < 0.05). On the other hand, western blot results indicated that the mRNA and protein expression levels of antiapoptotic gene Bcl2 was increased, the proapoptotic genes caspase-3 and Bax were decreased when cells were treatment with MSC-exo after hypoxia for 72 h (Fig. 6b). More importantly, Wnt5a siRNA transfection actually increased the cells apoptosis and the protein expression of Bax and Cleaved caspase 3, but decreased the expression of Bcl-2 as compared with MSC-exo group. These results provides a strong evidence that the inhibition of MSC-exo on hypoxia-induced PAEC apoptosis was through inhibition of Wnt5a expression.

### Effect of MSC-exo on hypoxia-induced PAEC migration and angiogenesis ability

**Fig. 7:**
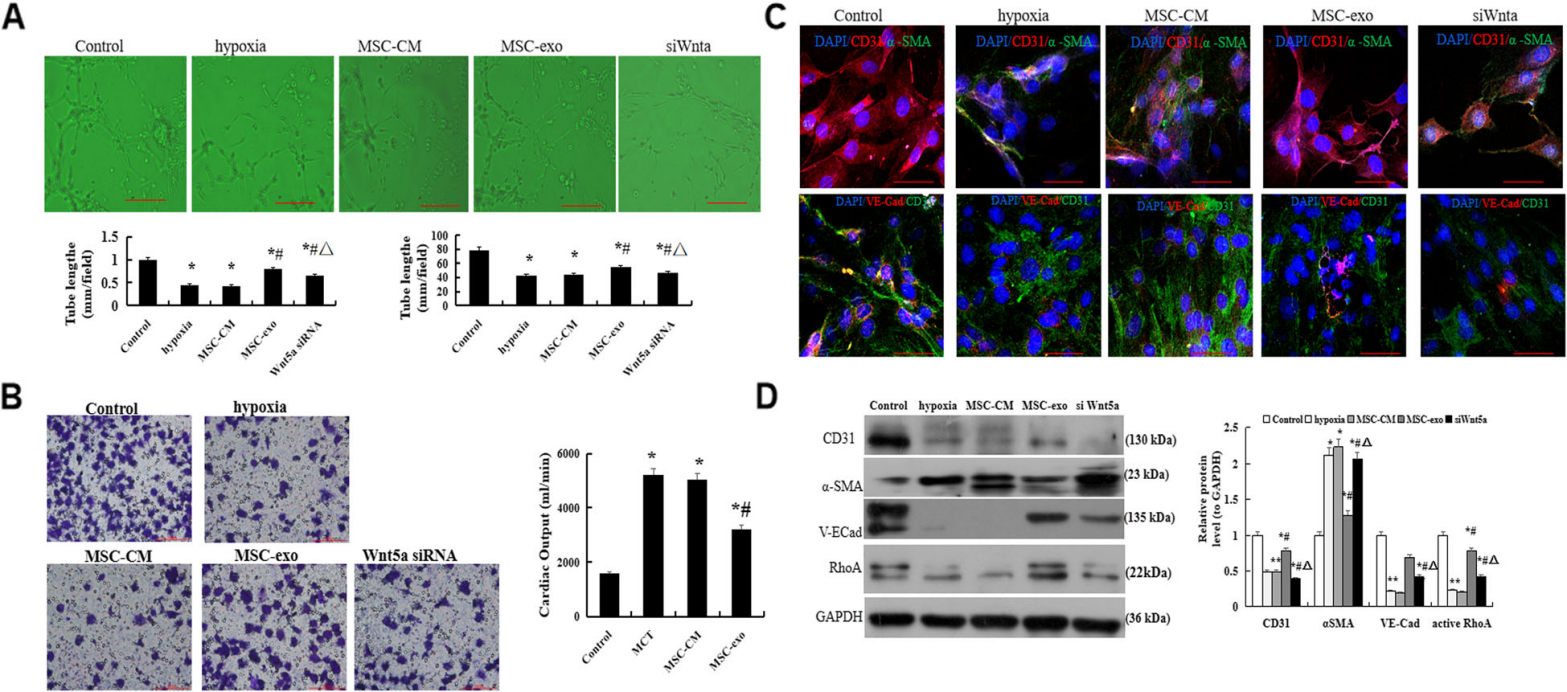
Effect of MSC-exo on hypoxia-induced PAEC angiogenesis, migration, EndMT and adhesion ability. **a** Tube formation on Matrigel-coated plates. **b** Immunofluorescence analysis of CD31, a-SMA and VE-Cadherin using a light microscope at a × 400 magnification. **b**. Transwell assays to observe migratory ability at a × 200 magnification. **c** Protein expression and comparative analysis of of CD31 and a-SMA (**d**) Protein expression and comparative analysis of RhoA in PAECs by Western blot. *n* = 3 times repeated; *P* < 0.05, one-way ANOVA followed by post hoc test; the data are present as mean ± SD; ^*^hypoxia or ^*^MSC-CM vs. control; ^#^MSC-exo vs. hypoxia group; ^*^ si Wnt5a vs. MSC-exo group. Scale bar = 100 μm

After PAEC were treatment with MSC-CM or MSC-exo in normoxia or 3% oxygen for 72 h, tube formation assay was performed using 48-well plates coated with Matrigel. Our results showed that the tube formation and capillary network branch points were significantly enhanced in MSC-exo treatment group as compared with hypoxic and MSC-CM groups, however, when the cells were transfection with Wnt5a siRNA, the tube formation and capillary network branch points were markedly reduced (*P* < 0.05, Fig. 7a).

Transwell assays were performed to observe the effect of MSC-exo on the migratory ability. As demonstrated in Fig. 7b, the migratory ability was lower in hypoxic damage group as compared with normal cells group, but it was obviously increased in treatment with MSC-exo group (*P* < 0.05).

### Effect of MSC-exo on hypoxia-induced EndMT and adhesion ability in PAECs

In order to observe the effect of MSC-exo on hypoxic-induced EndMT and cell adhesion, the protein expression of CD31, a-SMA and adherens junctions protein of VE-Cadherin were measured. Immunofluorescence results showed that the levels of CD31 and VE-Cadherin were higher, but α-SMA was lower when in MSC-exo group than that in hypoxic and MSC-CM groups (*P* < 0.05, Fig. 7c). Moreover, we detected the protein expression and RhoA by western blot, the results indicated that the protein expression of RhoA was significantly increased in MSC-exo group than that in hypoxic and MSC-CM groups (*P* < 0.05, Fig. 7d). However, these results indicated that the above changes could reverse after PAECs were transfection with Wnt5a siRNA. The above data suggested that the inhibition effect on EndMT and promotion adhesion ability of MSC-exo may be associated with regulation Wnt5a/RhoA signaling pathway.

### Effect of MSC-exo on hypoxia-induced proliferation of PAMSCs

**Fig. 8:**
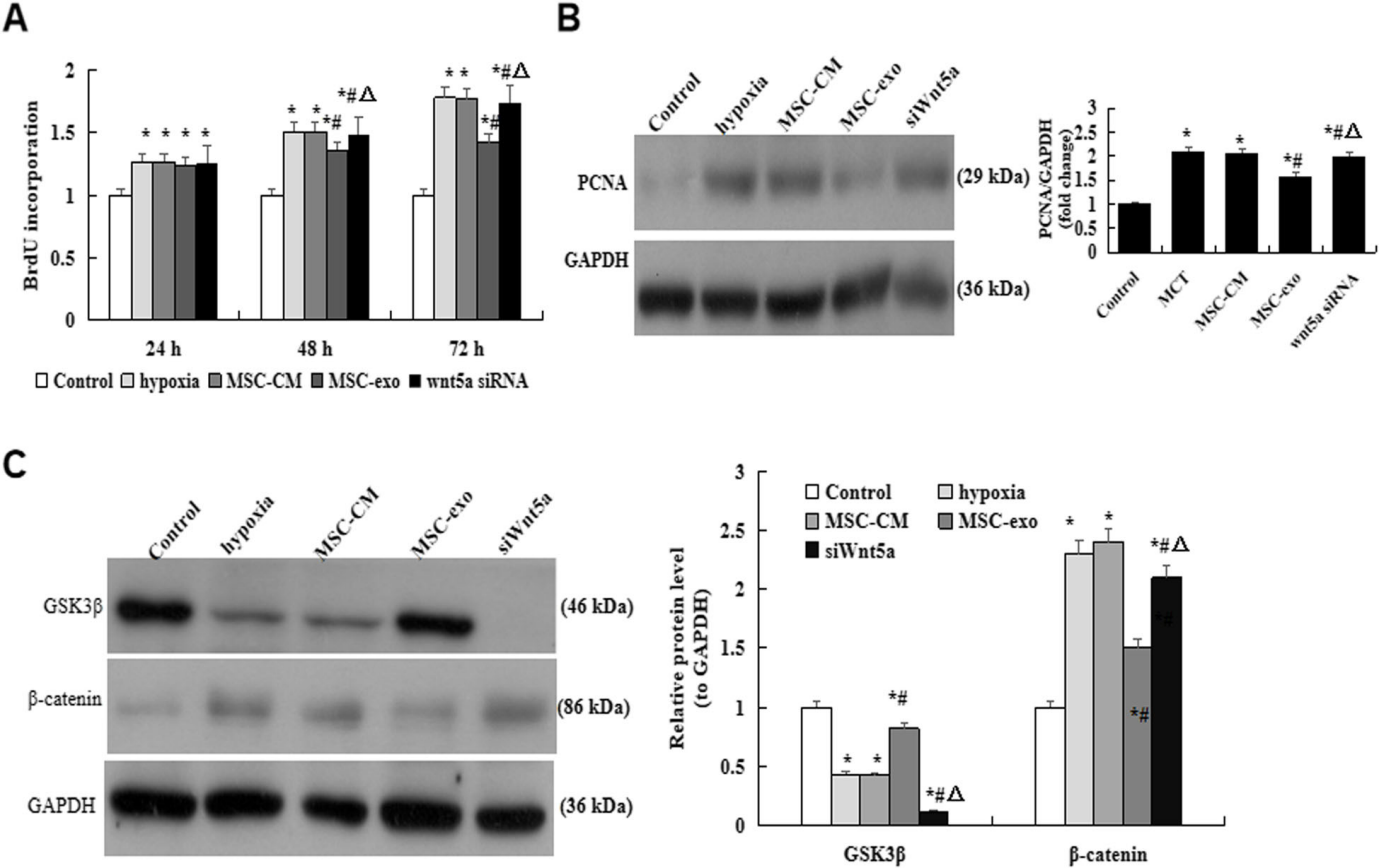
Effect of MSC-exo on hypoxia-induced proliferation of PAMSCs. **a** PAMSCs proliferation analysis by BrdU Cell Proliferation Assay. **b** Protein expression and comparative of PCNA. **c** Protein expression of GSK3β and β-catenin in PAMSCs after exposed to hypoxia for 72 h. **d** Comparative analysis of the protein expression of GSK3β and β-catenin. *n* = 3 times repeated; *P* < 0.05, one-way ANOVA followed by post hoc test; the data are present as mean ± SD; ^*^hypoxia or ^*^MSC-CM vs. control; ^#^MSC-exo vs. hypoxia group; ^*^ si Wnt5a vs. MSC-exo group

The effect of MSC-exo against hypoxia-PAMSC injury by classical determination of BrdU. Briefly, PASMC proliferation was measured when cells treatment with a hypoxia or normoxia condition in the presence or absence of MSC-exo for 24 h, 48 h and 72 h using the BrdU Cell Proliferation Assay Kit. Our indicated that the proliferation was significantly reduced in MSC-exo group as compared with hypoxic exposure for 48 h and 72 h groups (*P* < 0.05, Fig. 8a). The protein expression of PCNA was measured by western blot, which indicated that protein level of PCNA was obviously decreased in MSC-exo group as compared with hypoxic and MSC-CM groups (*P* < 0.05, Fig. 8b). On the other hand, the protein expression of GSK3β and β-catenin were also detected by western blot, the results showed that β-catenin was significantly decreased, GSK3β was significantly increased in MSC-exo group than that in hypoxic and MSC-exo groups (*P* < 0.05, Fig. 8c). More importantly, the above data were significantly reverse after PAMSCs were transfection with Wnt5a siRNA (*P* < 0.05).

## Discussion

Pulmonary arterial hypertension (PH) is a kind of refractory rare lung diseases, distal pulmonary arterial remodeling is the characteristic of it. The vascular remodeling in PH including pulmonary endothelial cells apoptosis and abnormal smooth muscle cells proliferation [22]. The pathogenesis of PH is not clear yet, therefore, so far there is still no effective prevention measures for it. Previous studies have suggested the exosomes isolated from mesenchymal stem cells (MSCs) has the potential to inhibition of vascular remodeling in PH, which is considered as a novel potential therapeutic approach for the treatment of PH [10, 11, 15]. However, the mechanism is not fully clear. In the present study, our data confirmed that injection of exosomes derived from human umbilical cord MSCs (hUCMSCs) could significantly inhibit the progression of MCT-induced PH vascular remodeling, reduce the degree of lung fibrosis and right ventricular hypertrophy. Moreover, our results demonstrated that MSC-exo could significantly reduced the percentage of fully muscularized vessels and the expression of α-SMA, but increase the expression of CD31 as compared with MCT and MSC-CM rats.

Wingless (Wnt) signaling pathway is divided into canonical signaling pathway and non-canonical signaling pathway. As a member of Wnt family, mounting evidence indicated that wnt5a involved in the pathogenesis of PH [13, 14, 23, 24], wnt5a was reported to have the role of regulating human endothelial cell proliferation and migration via noncanonical signaling [25] and inhibiting hypoxia-induced PASMC proliferation via suppression of of β1-catenin/cyclin D1 [26]. Recently studies showed that wnt5a could inhibition fibroblast proliferation and resistance to apoptosis in PH and could right heart failure following recovery from hypoxia, which maybe correlate with reduced pericyte coverage of small vessels [13, 14, 27]. Our present study demonstrated that the expression level of wnt5a was increased, but β-catenin and cyclin D1 were decreased in MSC-exo-treated group as compared with MCT and MSC-CM-treated groups. These results suggest that the inhibition mechanism of MSC-exo on PH pulmonary vascular is through regulation of Wnt5a/β-catenin and its target gene Cyclin D1.

To further observe the mechanism, hypoxia-injury cells model was established in the present study. The experiment results in vitro showed that when the cells were hypoxia exposure for 72 h, the expression of wnt5a were significantly decreased in PAECs and PAMSCs, however, it was significantly increased in MSC-exo group. Furthermore, we analyzed PAEC apoptosis migration, and vessel formation ability of PAECs under hypoxic conditions, the results indicated that the percentage of apoptotic cells and the expression levels of antiapoptotic gene Bcl2 were increased, but the expression levels of proapoptotic genes caspase-3 and Bax were decreased in MSC-exo group than hypoxia and MSC-CM groups. However, these data was markedly reversed when the cells were transfection with wnt5a siRNA. The results suggested that the suppression of MSC-exo on PH pulmonary vascular remodeling were associated with up-regulation wnt5a expression. Non-canonical Wnt signaling regulation cell migration and cell polarity, also controls sprouting angiogenesis and vascular remodeling [28, 29]. Wnt5a through regulating non-canonical Wnt signaling helps cells to move together by stabilizing vinculin at cell junctions and play an important role in endothelial cell migration [30]. RhoA could promote focal adhesion and regulates some cancer cell contractility, leading to cell migration, but blocking RhoA activity of RhoA could significantly inhibition the migration in wnt5a-induced cells [31, 32]. Therefore, it is possible that wnt5a-induced RhoA activation may participate in the regulation of endothelial cell migration. The present study results showed that MSC-exo could obviously enhance tube formation and migratory ability after PAECs exposed to hypoxia for 72 h. The potential role of EndMT in vascular remodeling and the fibrotic lung disease haas also been reported [33, 34] Further experiments found that the expression of CD31 and VE-Cadherin was significantly higher, but α-SMA was lower. However, when siRNA wnt5a transfection the cells, the above data were apparent reversal. Taken together, our present study confirmed that MSC-exo could suppress hypoxia-induced EndMT in PAECs, promote cell adhesion and contraction, the mechanism was through regulation of wnt5a signaling pathway.

Abnormal GSK3β signaling was recently implicated in various vascular- and fibro-proliferative diseases [35–37]. Previous reports [38, 39] showed that total and p-GSK3β remains increased and inactivated in MCT-induced PH lung tissue and MCT-PASMCs than in control, activation of GSK3β could reduction p-GSK3β levels and related with excessive proliferation of PASMCs in PH. GSK3β signaling may trigger the proliferative phenotype of PASMC, which indicated that GSK3β has a central role in the process of vascular remodeling. Thus, may be play a critical regulatory role in PH vascular remodeling, and can be seen a novel therapeutic opportunity for the treatment of PH. In the present study, our BrdU cell proliferation assay results showed that after treatment cell with MSC-exos for 48 h and 72 h, PAMSC proliferation ability has been restricted. However, the above changes were reversed when the cells were transfection with Wnt5a siRNA. Most importantly, the protein expression of β-catenin was significantly decreased, GSK3β was significantly decreased in MSC-exo group. Synthetically, our data provide a strong evidence for the association of Wnt5a related signaling with the effect of MSC-exo on MCT-PH and hypoxia-induced cells injury.

## Conclusion

In conclusion, findings from the present study demonstrated that MSC-exo injection could attenuate PH pulmonary vascular remodel. The experiment results showed that MSC-exo increased the expression of wnt5a further to regulation RhoA and GSK3β/β-catenin signaling pathway. Those can not be clarified in the present study and would need further investigations.

## Abbreviations

EndMT: Endothelial-to-mesenchymal transition
Exo: Exosomes
hUCMSCs: Human umbilical cord mesenchymal stem cells
LV + S: Left ventricular plus septal weight
MCT: Monocrotaline
PAEC: Pulmonary arterial endothelial cell
PASMC: Pulmonary arterial smooth muscle cells
PCNA: Proliferating cell nuclear antigen
PH: Pulmonary hypertension
RT-PCR: Real-time polymerase chain reaction
RVSP: Right ventricular systolic pressure
TEM: Transmission electron microscope
Wnt: Wingless
